# Elevated IL-6 and Tumor Necrosis Factor-α in Immune Checkpoint Inhibitor Myocarditis

**DOI:** 10.3390/diseases12050088

**Published:** 2024-05-03

**Authors:** Abdelrahman Ali, Rebecca Caldwell, Gaspar Pina, Noah Beinart, Garrett Jensen, Syed Wamique Yusuf, Efstratios Koutroumpakis, Ihab Hamzeh, Shaden Khalaf, Cezar Iliescu, Anita Deswal, Nicolas L. Palaskas

**Affiliations:** 1 Department of Cardiology, Division of Internal Medicine, The University of Texas MD Anderson Cancer Center, 1515 Holcombe Blvd, Unit 1451, Houston, TX 77030, USA; aali13@mdanderson.org (A.A.); rebecca.l.caldwell@uth.tmc.edu (R.C.); gaspar.pina@uth.tmc.edu (G.P.); nibeinart@mdanderson.org (N.B.); syusuf@mdanderson.org (S.W.Y.); ekoutroumpakis@mdanderson.org (E.K.); irhamzeh@mdanderson.org (I.H.); szkhalaf@mdanderson.org (S.K.); ciliescu@mdanderson.org (C.I.); adeswal@mdanderson.org (A.D.); 2 Texas A&M College of Medicine, Center for Genomics and Precision Medicine, Houston, TX 77030, USA; gjensen3@tamu.edu

**Keywords:** Immunotherapy, Myocarditis, Cytokines, Cardiotoxicity

## Abstract

Introduction: The impact of peripheral cytokine levels on the prognosis and treatment of immune checkpoint inhibitor (ICI) myocarditis has not been well studied. Objectives: This study aimed to identify cytokines that can prognosticate and direct the treatment of ICI myocarditis. Methods: This was a single-center, retrospective cohort study of patients with ICI myocarditis who had available peripheral cytokine levels between January 2011 and May 2022. Major adverse cardiovascular events (MACEs) were defined as a composite of heart failure with/without cardiogenic shock, arterial thrombosis, life-threatening arrhythmias, pulmonary embolism, and sudden cardiac death. Results: In total, 65 patients with ICI myocarditis had cytokine data available. Patients were mostly males (70%), with a mean age of 67.8 ± 12.7 years. Interleukin (IL)-6 and tumor necrosis factor-α (TNF-α) were the most common cytokines to be elevated with 48/65 (74%) of patients having a peak IL-6 above normal limits (>5 pg/mL) and 44/65 (68%) of patients with peak TNF-α above normal limits (>22 pg/mL). Patients with elevated peak IL-6 had similar 90-day mortality and MACE outcomes compared to those without (10.4% vs. 11.8%, *p* = 0.878 and 8.8% vs. 17.7%, *p* = 0.366, respectively). Similarly, those with elevated peak TNF-α had similar 90-day mortality and MACEs compared to those without (29.6% vs. 14.3%, *p* = 0.182 and 13.6% vs. 4.8%, *p* = 0.413, respectively). Kaplan–Meier survival analysis also showed that there was not a significant difference between MACE-free survival when comparing elevated and normal IL-6 and TNF-α levels (*p* = 0.182 and *p* = 0.118, respectively). MACEs and overall survival outcomes were similar between those who received infliximab and those who did not among all patients and those with elevated TNF-α (*p*-value 0.70 and 0.83, respectively). Conclusion: Peripheral blood levels of IL-6 and TNF-α are the most commonly elevated cytokines in patients with ICI myocarditis. However, their role in the prognostication and guidance of immunomodulatory treatment is currently limited.

## 1. Introduction

Immune checkpoint inhibitors (ICI) have greatly advanced cancer therapy over the last decade and improved overall survival for patients [1]. However, ICI can cause a myriad of immune-related adverse events including myocarditis, which can occur in up to 1–3% of patients receiving ICI [2]. Several diagnostic tests are used in combination to make a diagnosis of ICI myocarditis, including the clinical presentation, cardiac biomarkers, multimodality cardiac imaging, and endomyocardial biopsy (EMB) [3]. Even though it is an invasive procedure, EMB remains the gold standard for diagnosing myocarditis [4,5,6]. Typical histologic findings of ICI myocarditis on EMB samples show a predominantly CD8+ T cell infiltration into the myocardium [5]. Few preclinical and clinical studies have examined the pathophysiology behind ICI myocarditis [7,8,9]. It is suspected that antigenic mimicry between antigens on the cardiomyocyte such as troponin or myosin and tumor cells causes T cell activation and induction of an inflammatory cascade [10]. Tumor necrosis factor-α (TNF-α) is one of many cytokines released in this process and has been implicated as a driving factor for greater myocyte necrosis in the mouse model of viral myocarditis [11].

The clinical utility of monitoring cytokine levels for diagnostic and prognostic purposes has not been elucidated in patients with ICI myocarditis [12]. Additionally, utilizing cytokine inhibitors such as those targeting TNF-α (infliximab) and interleukin (IL)-6 (tocilizumab) in steroid-refractory ICI colitis has been found to mitigate symptoms and accelerate recovery [13,14]. Limited data exist about the role of cytokines in guiding therapy for patients presenting with ICI myocarditis [15]. Furthermore, infliximab use may increase the risk of cardiovascular death in patients with ICI myocarditis [16]. The aims of this study were to evaluate the prognostic significance of peripheral cytokine levels and to explore if cytokine levels can guide immunomodulatory treatment of ICI myocarditis.

## 2. Methods

### 2.1. Study Population

This was a single-center retrospective cohort study of patients with ICI myocarditis treated at MD Anderson Cancer Center in Houston, Texas, between January 2011 and May 2022. A query of the electronic medical record for a problem list including “myocarditis” was performed. Electronic medical records were then reviewed to identify patients with ICI myocarditis based on the certainty adjudication criteria detailed below. Patients with other causes of myocarditis such as viral/bacterial myocarditis were excluded from the study.

The study’s protocol received approval from the Institutional Review Board (IRB), and informed consent was waived due to the retrospective design of the research.

#### Laboratory Data Collection

Cardiac biomarkers (NTproBNP and Troponin T) were collected during the index evaluation for myocarditis. Serum cytokine levels were measured by two separate clinical laboratory improvement amendments (CLIA)-certified assays:

- Cytokine A panel—Interleukin (IL)-6, Interferon-γ (IFN-γ), Tumor Necrosis Factor-α (TNF-α)
- Cytokine B panel—(Send-out lab)-IL-2, IL-1β, IL-10, IL-12, IL-13, IL-17, IL-2 receptor, IL-4, IL-5, IL-8

Serum samples were collected using red-top tubes devoid of additives. The analysis of cytokine A was conducted employing an enzyme-linked immunosorbent assay (ELISA) based on Luminex technology. Cytokine B samples, in contrast, were transported on wet ice and subjected to analysis using the Milliplex multiplexing approach, which utilizes a fluorometric detection method based on Luminex-xMAP technology (Austin, Texas, USA). If cytokine levels were measured more than once, peak cytokine levels after myocarditis presentation were used for analysis.

### 2.2. Clinical Events Adjudication

ICI myocarditis was diagnosed using established certainty adjudication criteria [3]. Briefly, ICI myocarditis was classified into 3 different categories: definite, probable, and possible, based on EMB, laboratory, and imaging study results (Supplement S1) [3]. All EMB were obtained from the right ventricular septum with at least 5 samples collected during the same procedure. Experienced cardiac pathologists familiar with both heart transplant rejection and ICI myocarditis interpreted the EMB samples. Myocarditis severity was further classified into 4 different grades (1–4) based on American Society of Clinical Oncology clinical practice guidelines [16].

Major adverse cardiovascular events (MACEs) were defined as a composite of heart failure with/without cardiogenic shock, arterial thrombosis (myocardial infarction, stroke, lower limb ischemia), life-threatening arrhythmias (asystole, pulseless electrical activity, third-degree heart block, sustained ventricular tachycardia, ventricular fibrillation), pulmonary embolism, and sudden cardiac death. Patients were also evaluated for overlap neuromuscular irAEs, myositis and myasthenia gravis. The diagnosis of myositis and myasthenia gravis was made by a neurooncologist based on clinical criteria, blood laboratory testing, electromyography, and for some patients, quadriceps skeletal muscle biopsy.

### 2.3. Immunomodulatory Treatment

The treatment regimens used for ICI myocarditis were administered as per the healthcare provider’s clinical discretion. As per expert consensus guidelines, steroids are the mainstay of treatment for ICI myocarditis at our institution. Different strategies are used based on the treating physician’s preference for use of immunomodulatory treatment with either upfront concomitant use with steroids or only for those that are refractory to steroid therapy based on clinical deterioration and/or troponin level response.

### 2.4. Statistical Analysis

Baseline characteristics were interpreted as mean ± SD or median (IQR) for continuous variables and frequency for categorical variables, respectively. The normality of continuous variables was determined based on the visual evaluation of histograms and the Shapiro–Wilk test. Baseline characteristics were compared between those with normal TNF-α vs. those with elevated TNF-α using a 2-sample Student’s *t*-test or the Wilcoxon rank sum test for continuous variables and the chi-square or Fisher exact test for categorical variables, respectively. Kaplan–Meier survival analysis was used to estimate the survival free from MACEs. Overall survival time was defined as the time interval from initial healthcare contact with suspected ICI myocarditis to death from any cause. A *p*-value <0.05 indicated statistical significance. STATA version 17.0 was used for data analysis.

## 3. Results

**Figure 1:**
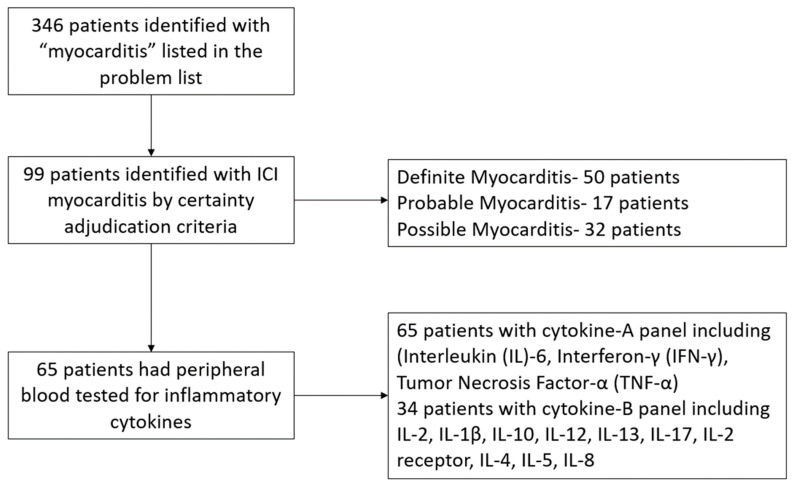
Study flow diagram.

The study cohort included 99 patients with adjudicated myocarditis of whom 65 (66%) had data available for cytokines (Figure 1). In the overall cohort, the mean age was 67.8 ± 12.7 years, 70% were males, and 83% were Caucasians. The most common cancer in the study population was genitourinary (35%) followed by melanoma (22%) and lung cancer (17%). The majority of patients received programmed death-1 (PD1) inhibitors (79%) followed by programmed death ligand-1 (PD-L1) inhibitors (13%). Based on the certainty adjudication criteria for myocarditis detailed in the methods Section, 50% of patients had definite myocarditis, 17% had probable myocarditis, and 32% had possible myocarditis. Overlap neuromuscular immune-related adverse events were observed in 45 patients (46%) with most having concomitant myocarditis, myositis, and myasthenia gravis (22 patients, 49%) followed by concomitant myocarditis and myositis (19 patients, 42%) (Table 1).

The median value for peak Troponin T was 639.5 ng/mL (IQR: 229–1585), while the median value for peak NTproBNP was 1678 pg/mL (IQR: 455–4326) (Table 1). The majority of patients had elevated levels of IL-6 and TNF-α greater than the upper limit of normal levels (74% and 68%, respectively). Only 26% of patients had elevations in IFN-γ (Table 2). The median values for patients with elevated IL-6 and elevated TNF-α were 27.5 pg/mL (IQR 18, 83) and 36 pg/mL (IQR 30, 68), respectively, while the median value of IFN-γ was 10 pg/mL (IQR 8, 17) (Table 2). There was no observed correlation between the histologic grade of myocarditis and the levels of IL-6 and TNF-α (Supplement S2). There were 34 patients (34%) who had the expanded cytokine B panel. In the cytokine B panel, the most common elevations were for IL-2 receptor and IL-2 (62% and 20%, respectively). None of the patients had elevation in IL-1β (Table 2). The median time to serum collection for cytokine analysis was 2 days (IQR 0, 6 days) from index admission day or initial outpatient evaluation of ICI myocarditis.

Treatment regimens varied with 81% of patients receiving steroids and 61% receiving immunomodulator(s) in addition to steroids. One of the most common secondary therapies received in addition to steroids was plasmapheresis (42%) followed by TNF-α inhibition with infliximab in 22% of patients. None of the patients who received infliximab developed worsening heart failure after infliximab administration. None of the patients received IL-6 inhibition with tocilizumab; therefore, much of the analysis focused on comparing those with elevated TNF-α to those without TNF-α elevation since 22% of patients received TNF-α inhibition.

There were no statistically significant differences in demographics, comorbidities, or ICI class received between patients with normal and elevated TNF-α. However, it was noted that there were significantly more patients with higher grade toxicity in the elevated TNF-α group compared to those with normal TNF-α (Table 1). In those with elevated TNF-α, there were 20 patients (46%) with grade 3 and 8 patients (18%) with grade 4 compared to only 7 patients (33%) with grade 3 and none with grade 4 in the normal TNF-α group (Table 1). There was no statistically significant difference in the treatments received between patients with normal TNF-α vs. those with elevated TNF-α (Table 3).

Death at 90 days from initial presentation with myocarditis occurred in 23 patients (23%). MACEs occurred in 13 patients (13%) with heart failure being the most common followed by arrhythmia (5%) and sudden cardiac death (4%) (Table 4). Although mortality was numerically higher in the elevated IL-6 group (16.7% vs. 7.3%, *p* = 0.24) and the elevated TNF-α, the differences in mortality between patients with elevated cytokines vs. those without were not statistically significant (Table 4). There was also no statistically significant difference in the composite outcome of MACEs in patients with elevated IL-6 vs. those without (8.3% vs. 17.7%, *p* = 0.37) and elevated TNF-α vs. those without (13.6% vs. 4.8%, *p* = 0.41) (Table 4). In patients with an elevated TNF-α > 22 and stratified by sex, there was no difference in 90-day mortality (33.3% in males vs. 25% in females, *p* = 0.59) or MACEs (18.2% vs. 0, *p* = 0.11) (Supplement S3). Outcomes stratified by sex for IL-6 > 5 pg/mL are detailed in Supplement S4. 

**Figure 2:**
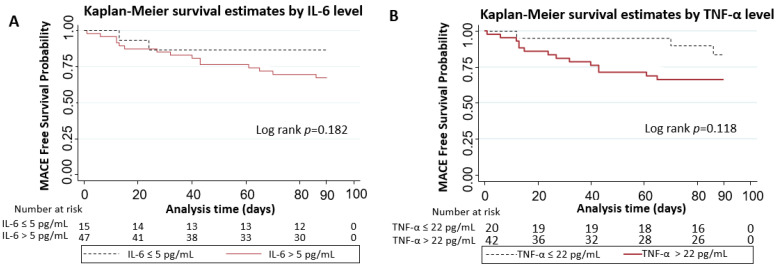
Comparison of MACE-free survival in patients with (**A**) IL-6 levels above the normal cut-off level of 5 pg/mL and those with normal IL-6 levels and (**B**) TNF-α levels above the normal cut-off level of 22 pg/mL vs. those with normal TNF-α levels.

**Figure 3:**
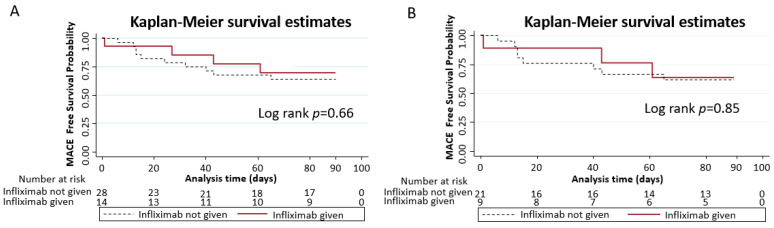
(**A**) The administration of infliximab showed similar MACE-free survival among patients with elevated TNF-α > 22 pg/mL. (**B**) The administration of infliximab showed similar MACE-free survival among patients with elevated TNF-α > 30 pg/mL.

Kaplan–Meier analysis showed that patients with IL-6 levels above the normal cut-off level of 5 pg/mL and TNF-α levels above the normal cut-off level of 22 pg/mL had comparable MACE-free survival to those with normal IL-6 levels and TNF-α levels, respectively (Log-rank *p* = 0.18 and *p* = 0.12, respectively) (Figure 2). The administration of infliximab showed similar MACE-free survival between all patients and in a subgroup of those with elevated TNF-α (*p*-value 0.66 and 0.85, respectively) (Figure 3). None of the patients received tocilizumab or any other IL-6 inhibition and, therefore, a comparison of outcomes with therapy targeted at IL-6 was not able to be performed.

## 4. Discussion

Our cohort study is the largest to examine the relationship between serum peripheral blood cytokine levels and ICI myocarditis outcomes. The results show that the most common cytokines to be elevated in the peripheral blood during ICI myocarditis are IL-6 and TNF-α with a surprising minority of patients with IFN-γ elevation and none with IL-1β elevation. Although there were numerically higher MACE events and 90-day mortality in patients with elevated TNF-α levels, the differences were not statistically significant. However, the administration of infliximab showed similar MACE-free and overall survival between all patients and those with elevated TNF-α levels.

Matsumori et al. found an increase in inflammatory cytokines such as TNF-α, interleukin (IL) 1-a/β, and granulocyte-macrophage colony-stimulating factor (GM-CSF) in patients with non-ICI-related myocarditis compared to other cardiac conditions [17]. The study suggested that TNF-α plays a role in the pathogenesis of myocardial injury and it might be contributing to the decline in cardiac function. A case series of ICI myocarditis patients published by Tsuruda et al. showed an increase in the following inflammatory cytokines: IL-8, monocyte chemotactic and activating factor, and granulocyte-macrophage colony-stimulating factor (GM-CSF) in one case. IL-8 was predominantly increased in a second case, while the third case had elevations in IL-6, IL-8, GM-CSF, and IFN-γ. It was also reported that TNF-α was elevated during the recovery phase of the third case [15]. Surprisingly, only 26% of patients in our cohort had elevation in IFN-γ. Given that several studies have described CD8+ cytotoxic T cell infiltration in the myocardium of patients with ICI myocarditis [5,18], it would be expected that IFN-γ is involved given that the differentiation of naïve CD8+ T cells into cytotoxic T cells requires IFN-γ [19,20]. One possible explanation for this discrepancy is that we evaluated peripheral blood cytokine levels, which may not reflect the myocardial microenvironment cytokine levels for which IFN-γ works through autocrine signaling [20].

In another study examining melanoma patients receiving ipilimumab, low IL-6 was found to be of prognostic value and was predictive of which patients developed irAEs. However, none of the patients in that study developed myocarditis [17]. Moreover, in a limited study which included only patients with melanoma, Head et al. showed that pre-treatment values of TNF-α and IFN-α were associated with higher grades of irAEs, but myocarditis was again not reported [3]. In our cohort, both TNF-α and IL-6 were elevated during the presentation with ICI myocarditis (Table 2). However, we were not able to establish the prognostic value of elevated TNF-α or IL-6 above the upper limit of normal for MACE events or overall mortality.

Post-marketing data with TNF-α inhibitors describe congestive heart failure or worsening existing heart failure after TNF-α inhibitor use [21,22], and it has also been reported to be associated with hypersensitivity myocarditis [23]. Additionally, a randomized, double-blind, placebo-controlled trial which evaluated infliximab use (at 5 mg/kg and 10 mg/kg doses) in patients with moderate to severe heart failure showed an increased risk of death from any cause or heart failure hospitalization at the 10 mg/kg dose compared to a placebo. Increased mortality and heart failure were not observed for patients receiving the 5 mg/kg infliximab dose. Infliximab is commonly used for ulcerative colitis, rheumatoid arthritis, and psoriasis. All patients in our cohort who received infliximab received the 5 mg/kg dose [24]. A case–control study by Cautela et al. examined intensified immunosuppressive therapy (IIST) for patients presenting with ICI myocarditis and reported that patients (n = 8) treated with infliximab were more likely to die from cardiovascular causes (OR, 12.0; *p* = 0.005), which is contrary to findings in our study cohort (n = 23). However, cytokine data were not available in that study [16]. Though it was not statistically significant, there was a higher rate of infliximab utilization in our cohort, especially in patients with elevated TNF-α, although this may have been related to more patients with elevated TNF-α having higher grade myocarditis and thus requiring immunomodulatory treatment beyond steroids. The number of patients with grade 3 or 4 cardiotoxicity was too small for a comparison of outcomes for those receiving infliximab versus not. More recently, in a small case series, infliximab administration in patients with ICI myocarditis did not lead to increased decompensated heart failure and cardiogenic shock [25]. The American Society of Clinical Oncology integrated the use of infliximab in their guideline update, especially in patients without a rapid response to high-dose steroids [26].

Tocilizumab, an IL-6 inhibitor used in autoimmune diseases like rheumatoid arthritis and giant cell arteritis, was not administered to our patient cohort, thus precluding its evaluation in those with elevated IL-6 levels. Tocilizumab is also used in cytokine release syndrome due to chimeric antigen receptor T cell therapy (CAR-T), but there are only limited reports in use for ICI myocarditis. It is currently being evaluated as an upfront treatment with ICI to prevent all irAEs, not specifically ICI myocarditis (NCT04940299).

Previous studies have shown the risk of developing ICI myocarditis to be less than <1%. However, it carries a high mortality rate of up to 25–50% and more recent prospective studies have suggested a higher incidence of myocarditis [27,28,29]. In our study, the mortality rate within the first 90 days was 23%, which is consistent with a meta-analysis by Rubio et al., in which the rate of ICI myocarditis was 0.72% and the mortality was 26.6% [30] and another small cohort study of 30 patients which reported a mortality of 27% [31]. In our cohort, MACE events were driven by heart failure and arrhythmias, which are also consistent with prior studies [2,31] such as the one by Mahmood et al. in which heart failure was observed in 42% of the study cohort of ICI myocarditis [2].

## 5. Limitations

Our study is a single-center retrospective study and the results may not be generalizable to other centers along with the fact that results may be biased by the treatments that patients received based on severity of disease and physician preferences. Given that the MD Anderson Cancer Center is a tertiary referral center, some patients may have had other clinical events that were not captured due to patients seeking treatment at other healthcare facilities. Further, the sample size was limited with a small number of MACE events, although our cohort included patients with various types of cancer and not exclusively melanoma as was the case with prior reports.

## 6. Future Implications

As previously alluded to, various inflammatory cytokines are involved in the pathogenesis of myocarditis and are dependent on its etiology. In our study, we showed that TNF-α and IL-6 are elevated in patients presenting with ICI myocarditis but have limited value in prognostication and guiding treatment. This may be due to serum peripheral cytokine levels not reflecting the effect of cytokines at the specific tissue level, such as within the myocardium. Further studies are needed to identify serum peripheral cytokine levels that correlate with cytokine levels at the site of tissue inflammation.

## 7. Conclusions

Our study demonstrates that although IL-6 and TNF-α are commonly elevated in patients with ICI myocarditis, they did not significantly predict MACEs or 90-day mortality. These findings cast doubt on the prognostic value of peripheral cytokine levels, particularly TNF-α, in predicting outcomes for ICI myocarditis. Additionally, the use of the TNF-α inhibitor infliximab did not lead to different survival outcomes, suggesting a complex interplay of immunological responses in affected patients.

This highlights the need for further research to elucidate the specific roles of cytokines directly within myocardial tissue rather than solely in peripheral blood. Future studies should aim to correlate tissue-specific cytokine profiles with clinical outcomes to enhance treatment precision and prognostic accuracy in ICI myocarditis. This might enable the development of more effective, targeted therapeutic strategies, potentially improving patient management and outcomes.

## Figures and Tables

**Table 1:** Demographics and clinical characteristics of the entire study cohort and stratified by normal versus abnormal TNF-α levels.

	Total Cohort (n = 99)	Peak TNF-α ≤ 22 pg/mL (n = 21)	Peak TNF-α > 22 pg/mL (n = 44)	*p*-Value, Comparing Peak TNF-α Groups
Age (years), mean ± SD	67.8 ± 12.7	69.6 ± 13.2	68.1 ± 12.9	0.66
Male sex, n (%)	69 (70.0)	15 (71.4)	32 (72.7)	0.85
Race, n (%)				0.75
Caucasian	82 (82.8)	19 (90.5)	37 (84.1)	
African American	6 (6.1)	-	3 (6.8)	
Asian	4 (4.0)	1 (4.8)	-	
Other	7 (7.1)	1(4.8)	4 (9.1)	
Hypertension, n (%)	75 (75.6)	18 (85.7)	34 (77.3)	0.65
Hyperlipidemia, n (%)	53 (53.5)	8 (38.1)	27 (61.4)	0.13
Diabetes, n (%)	24 (24.2)	4 (19.0)	10 (22.7)	0.97
Coronary Artery Disease, n (%)	25 (25.3)	6 (28.6)	11 (25)	0.99
Heart failure, n (%)	8 (8.1)	1 (4.8)	2 (4.5)	0.59
Tobacco history, n (%)	41 (41.4)	11 (52.4)	19 (43.2)	0.67
Cancer types, n (%)				0.88
Genitourinary	35 (35.3)	12 (57.1)	21 (47.8)	
Melanoma	22 (22.2)	4 (19.0)	9 (20.4)	
Lung	17 (17.2)	-	6 (13.6)	
Others	25 (25.3)	5 (23.8)	8 (18.1)	
ICI type *, n (%)				0.36
CTLA-4	5 (5.1)	-	-	
PD-1	78 (78.8)	18 (85.7)	39 (88.6)	
PD-L1	13 (13.1)	2 (9.5)	3 (6.8)	
PD-1 and CTLA-4	2 (2.0)	-	2 (4.6)	
PD-1 and LAG3	1 (1.0)	1 (4.8)	-	
Myocarditis classification, n (%)				0.20
Definite	50 (50.5)	9 (42.9)	27 (61.4)	
Probable	17 (17.2)	1 (4.8)	4 (9.1)	
Possible	32 (32.3)	11 (52.4)	13 (29.6)	
Cardiotox grade, n (%)				0.04
1	14 (14.1)	3 (14.3)	6 (13.6)	
2	35 (35.4)	11 (52.4)	10 (22.7)	
3	38 (38.4)	7 (33.3)	20 (45.5)	
4	12 (12.1)	-	8 (18.2)	
Any Concomitant Neuromuscular Immunotoxicity, n (%)	45 (46.0)	9 (42.9)	25 (56.8)	0.29
Types of Neuromuscular Toxicity in those with concomitant neuromuscular toxicity, n (%)				
Myositis	19/45 (42.2)	6/9 (66.7)	9/25 (36.0)	
Myasthenia Gravis	2/45 (4.4)	1/9 (11.1)	1/25 (4.0)	
Guillain Barre	2/45 (2.0)	-	1/25 (4.0)	
Myositis and Myasthenia Gravis	22/45 (48.9)	2/9 (22.2)	14/25 (56.0)	
Laboratory values				
NTproBNP (pg/mL), median (IQR)	1678 (455, 4326)	1360 (342, 2112)	2301 (570, 4727)	0.15
Troponin T (ng/mL), median (IQR)	639.5 (229–1585)	503 (177, 1292)	754.5 (419, 2079.5)	0.12

* CTLA-4: Cytotoxic T lymphocyte-associated antigen-4; PD-1: Programmed cell death-1; PD-L1: Programmed cell death ligand-1; LAG3: Lymphocyte activation gene 3.

**Table 2:** Cytokine levels and number of patients with abnormal cytokine levels in the entire cohort.

	Number of Patients with Elevation/Number of Patients with Levels Measured (% Positive) Median (Interquartile Range)
TNF-α normal ≤ 22 pg/mL	44/65 (67.7) 36 (30–68)
IFN-γ, Normal ≤ 5 pg/mL	17/65 (26.2) 10 (8–17)
IL-6, Normal ≤ 5 pg/mL	48/65 (73.8) 27.5 (18–83)
IL-2, Normal ≤ 12 pg/mL	7/34 (20.6) 12 (5–66)
IL-1β, Normal ≤ 36 pg/mL	0/34 (0.0) 10 (7–10.1)
IL-10, Normal ≤ 18 pg/mL	6/34 (17.7) 10.85 (6–16)
IL-12, Normal ≤ 6 pg/mL	2/34 (5.9) -
IL-13, Normal ≤ 5 pg/mL	4/34 (11.8) 11.5 (5.5–35.7)
IL-17, Normal ≤ 13 pg/mL	3/34 (8.8) 38.9 (29–72)
IL-2 receptor, Normal ≤ 1033 pg/mL	21/34 (61.8) 1476 (869–2167)
IL-4, Normal ≤ 5 pg/mL	2/34 (5.9) -
IL-5, Normal ≤ 5 pg/mL	1/34 (2.9) -
IL-8, Normal ≤ 5 pg/mL	1/34 (2.9) -

**Table 3:** Immunomodulatory management of myocarditis in the entire cohort and stratified by normal versus abnormal TNF-α level.

	Total Cohort (n = 99)	PeakTNF-α ≤ 22 pg/mL (n = 21)	Peak TNF-α > 22 pg/mL (n = 44)	*p*-Value, Comparing Peak TNF-α Groups
Types of Immunosuppression, n (%)				
Steroids	81 (81.8)	19 (90.5)	39 (88.6)	0.84
2. Immunomodulators	61 (75.3)	14 (66.7	32 (72.7)	0.31
Immunomodulators, n (%)				
1. Abatacept	2 (2.0)	-	2 (4.5)	1.00
2. Infliximab	23 (23.2)	6 (28.6)	16 (36.4)	0.54
3. IVIG	23 (23.2)	2 (9.5)	11 (25.0)	0.15
4. Mycophenolate	12 (12.1)	2 (9.5)	4 (9.1)	0.96
5. Plasmapheresis	42 (42.4)	10 (47.6)	27 (61.4)	0.26
6. Rituximab	21 (21.2)	7 (33.3)	13 (29.5)	0.76
7. Tacrolimus or cyclosporine	2 (2.0)	1 (4.8)	-	0.98

**Table 4:** Overall survival and MACE outcomes categorized by IL-6 and TNF-α levels.

Outcomes	Total Cohort (n = 99)	Peak IL-6 ≤ 5 pg/mL (n = 17)	Peak IL-6 > 5 pg/mL (n = 48)	*p*-Value, Comparing Peak IL-6 Groups	Peak TNF-α ≤ 22 pg/mL (n = 21)	Peak TNF-α > 22 pg/mL (n = 44)	*p*-Value, Comparing Peak TNF-α Groups
90-day mortality, n (%)	23 (23.2)	2 (11.8)	5 (10.4)	0.88	3 (14.3)	13 (29.6)	0.18
MACEs, n (%)	13 (13.1)	3 (17.7)	4 (8.3)	0.37	1 (4.8)	6 (13.6)	0.41
Heart failure	8 (8.1)	2 (11.7)	2 (4.2)	0.28	1 (4.8)	3 (6.8)	1.00
Arterial thrombosis	1 (1.01)	-	-	-	-	-	-
Arrhythmia	5 (5.1)	1 (5.9)	3 (6.3)	1.00	-	4 (9.1)	0.30
Pulmonary embolism	1 (1.01)	1 (5.9)	-	0.26	-	1 (2.3)	1.00
Sudden cardiac death	4 (4.0)	-	2 (4.2)	1.00	-	2 (4.6)	1.00

## Data Availability

The data from this study can be provided upon request to the corresponding author, due to considerations of privacy and legality.

## Supplementary Materials

The following supporting information can be downloaded at: https://www.mdpi.com/article/10.3390/diseases12050088/s1, Supplement S1: Certainty Adjudication Criteria as per criteria from Bonaca et al. [3]; Supplement S2: TNF-α and IL-6 levels compared to histologic grade of myocarditis; Supplement S3: Outcomes in patients with peak TNF-α > 22 pg/mL stratified by sex; Supplement S4: Outcomes in patients with peak IL-6 > 5 pg/mL stratified by sex.
