# Supplementary material for: Elevated IL-6 and Tumor Necrosis Factor-α in Immune Checkpoint Inhibitor Myocarditis
Source: Diseases. 2024 May 3;12(5):88. doi: 10.3390/diseases12050088 (PMC11120148; doi:10.3390/diseases12050088)
Supplement: Supplementary file 1 [file diseases-12-00088-s001.zip › diseases-2952185-supplementary.pdf]

Supplement S1- Certainty Adjudication Criteria as per criteria from Bonaca et al[3].

1. Definite myocarditis: the presence of at least one of the following:
    a. Pathology consistent with myocarditis.
    b. Diagnostic CMR, the clinical syndrome of myocarditis, and positive biomarker or ECG.
    c. Echocardiography with wall motion abnormality, the clinical syndrome of myocarditis, positive biomarker, positive ECG, and negative angiography for CAD.
2. Probable myocarditis:
    a. Diagnostic CMR without clinical syndrome of myocarditis, positive ECG, or positive biomarker, OR
    b. Suggestive CMR with one of the following:
        i. Clinical syndrome of myocarditis.
        ii. Positive ECG.
        iii. Positive biomarker, OR
    c. Echocardiography with wall motion abnormality and the clinical syndrome of myocarditis with either positive ECG or biomarker, OR
    d. Clinical syndrome of myocarditis with positron emission tomography scan evidence and no alternative diagnosis.
3. Possible myocarditis:
    a. Suggestive CMR without clinical syndrome of myocarditis, positive ECG, or positive biomarker, OR
    b. Echocardiography with wall motion abnormality and the clinical syndrome of myocarditis or positive ECG, OR
    c. Elevated biomarker with the clinical syndrome of myocarditis or positive ECG and no alternative diagnosis.

Supplement S2- TNF-α and IL-6 levels compared to histologic grade of myocarditis

	No biopsy	Grade 0[ǂ]	Grade 1A[ǂ]	Grade 1B[ǂ]	Grade 2[ǂ]	p-value*
TNF-α >22pg/mL, % (Positive/total)	64 (23/36)	100 (2/2)	67 (4/6)	75 (3/4)	71 (12/17)	0.825
TNF-α level if positive, median [IQR] (number of observations)	36 [28,54] (23)	77.5 [38,117] (2)	92 [33,182] (4)	75 [34, 176] (3)	35 [31.5,53] (12)	0.306
IL-6 >5 pg/mL percentage (Positive/total)	72 (26/36)	50 (1/2)	83 (5/6)	50 (2/4)	82 (14/17)	0.429
IL-6 level if positive Median [IQR] (number of observations)	41.5 [20,97] (26)	39 [39,39] (1)	21 [20,23] (5)	10 [8,12] (2)	30 [16,116] (14)	0.194

TNF-α: Tumor Necrosis Factor α, IL-6: Interleukin-6, IQR: Interquartile Range

* Calculated with $\chi 2$ test or Kruskal Wallis

ǂ Grading as per histologic criteria previously published[5]

Supplement S3. Outcomes in Patients with Peak TNF-α>22 pg/mL stratified by sex.

Outcomes	Females<br>Peak TNF-α > 22 (pg/mL)<br>(n= 12 /18)	Males<br>Peak TNF-α > 22 (pg/mL)<br>(n= 33 /47)	P-value
**90-day mortality, n (%)**	3/12 (25%)	11/33 (33.3%)	0.59
**MACE, n (%)**<br><br>**Heart failure**<br>**Pulmonary embolism**<br>**Sudden cardiac death**<br>**Arterial thrombosis**<br>**Arrhythmia**	**0**<br>-<br>-<br>-<br>-<br>-	6/33 (18.2%)<br>3 (9.4%)<br>1 (3.1%)<br>2 (6.3%)<br>0<br>3 (9.4%)	0.11

Supplement S4. Outcomes in Patients with Peak IL-6 > 5 pg/mL stratified by sex.

Outcomes	Females<br>Peak IL-6 >5 (pg/mL)<br>(n=14/18)	Males<br>Peak IL-6 >5 (pg/mL)<br>(n=31/47)	P-value
**90-day mortality, n (%)**	3/15 (20%)	10/33 (30.3%)	0.46
**MACE, n (%)**<br><br>**Heart failure**<br>**Pulmonary embolism**<br>**Sudden cardiac death**<br>**Arterial thrombosis**<br>**Arrhythmia**	0<br>-<br>-<br>-<br>-<br>-	4/31 (12.9%)<br>2 (6.5%)<br>0<br>2 (6.5%)<br>0<br>3 (9.7%)	0.29
